# Motivation towards medical career choice and academic performance in Latin American medical students: A cross-sectional study

**DOI:** 10.1371/journal.pone.0205674

**Published:** 2018-10-18

**Authors:** J. Smith Torres-Roman, Yuridia Cruz-Avila, Karina Suarez-Osorio, Miguel Ángel Arce-Huamaní, Alejandra Menez-Sanchez, Telmo Raúl Aveiro-Róbalo, Christian R. Mejia, Eloy F. Ruiz

**Affiliations:** 1 Faculty of Medicine, Universidad Nacional San Luis Gonzaga, Ica, Perú; 2 Faculty of Medicine, Universidad Nacional Autónoma de Honduras, Tegucigalpa, Honduras; 3 Faculty of Medicine, Universidad de los Andes, Mérida, Venezuela; 4 Faculty of Medicine, Universidad Autónoma del Estado de México, Toluca de Lerdo, México; 5 Faculty of Medicine, Universidad del Pacífico Privada, Asunción, Paraguay; 6 School of Medicine, Universidad Continental, Huancayo, Perú; 7 CONEVID, Unidad de Conocimiento y Evidencia, Universidad Peruana Cayetano Heredia, Lima, Peru; B P Koirala Institute of Health Sciences, NEPAL

## Abstract

**Introduction:**

Motivation in medical students is positively associated with learning strategies. However, the evidence of a direct relationship between motivation and performance is vague. The objective of this study is to determine if the motivation that pushed students to choose the medical career is associated with their academic performance during their university years.

**Methods:**

The study was conducted in 4,290 medical students from 10 countries in Latin America. The “Attribution Scale of General Achievement Motivation” was used to evaluate their general performance. The “Medical motivation Scale” test was used to measure social, altruist, economic, and prestige motivators. For statistical analyses, frequencies and percentages were described, and generalized linear models were used to establish statistical associations.

**Results:**

Fifty percent of the students surveyed were females and the mean student age was 21 years old. This study showed that male students had a higher social/altruist motivation (PR:1.11,95%CI: 1.03–1.18; p<0,01) than females. Those who had familial pressure had a lower social/altruist motivation (PR:0.17,95%CI:0.08–0.36; p<0,001). The positive vocational test was associated with a higher social/altruist motivation (PR:1.85,95%CI:1.03–3.30; p<0,05). Moreover, good grades at school were related with a higher economical/prestige motivation (PR:1.39,95%CI:1.05–1.83; p<0,05), but lower social/altruist motivation (PR:0.85,95%CI:0.74–0.98; p<0,05) and academic performance (PR:0.63,95%CI:0.50–0.79; p<0,001). We found a higher frequency in the general motivation was associated to a lowest social/altruist motivation (PR: 0.57; CI95%: 0.46–0.70; p<0.001), and that it increased according to the year of study (PR: 1.15; CI95%: 1.03–1.28; p:0.013) and was higher when pressure by the family was present (PR: 1.36; CI95%: 1.17–1.59; p<0.001).

**Conclusion:**

This study indicated that male medical students and having a positive vocational test were associated with a higher social/altruist motivation. Conversely, those who had familial pressure and good grades at school had a lower social/altruist motivation. Is necessary to conduct further studies that assess other factors related to motivation as demographics, personality, and learning styles.

## Introduction

In the past years, it has been found that student’s motivation is positively associated with learning strategies and academic performance[1–7]. Thus, we may asume that increases in a student’s motivation could lead to improvements in academic persistence, performance, as well as their use of different learning techniques [8].

However, the evidence of a direct relationship between motivation and performance is still barely known [9]. Motivation is determined by intrinsic factors (social/altruist), such as the interest of helping others and reaching personal growth, that may influence a student’s motivation during the academic stage [3,4,10]. Extrinsic factors (economical/prestige), such as the image expected to show to the society or salary that will be received in the future may also influence a student’s motivation [5,11,12]. Therefore, the influence of these factors will be reflected in the academic performance of the student through their achievements [3–5].

Motivation of medical students is usually different than students of other careers, given their pivotal role of commitment to proper decision-making in healthcare. For this reason, the admission process to medical careers usually consists on a high and strict selection based on aptitude and knowledge tests [13].

There is a high possibility that the selected students are the most prepared to attend medical school[13]. Nevertheless, the selection processes based solely on aptitude tests do not contemplate if the students have a better vocation for the career or if they have the social skills necessary for a humanitarian management with patients [14].

This study aims to determine if the motivation that pushed Latin American students to choose the medical career is associated with their academic performance during their medical studies.

## Methods

### Study design

A cross-sectional, analytic, observational and multi-center study was conducted.

### Location and time

This study was conducted between March and June of 2016 on 4,615 medical students from first to sixth year, belonging to ten Latin American countries. All universities of this study selection to students with strict selection based on aptitude and knowledge tests. The universities included were: Universidad Nacional de Tucumán (Argentina), Universidad Mayor de San Andrés (Bolivia), Universidad Autónoma de Bucaramanga (Colombia), Universidad Nacional de Chimborazo (Ecuador), Universidad Nacional Autónoma de Honduras (Honduras), Universidad Autónoma del Estado de México (Mexico), Universidad de Panamá (Panama), Universidad del Pacífico Privada (Paraguay), Universidad Nacional San Luis Gonzaga (Perú) and Universidad de los Andes (Venezuela).

### Sampling

The minimum sample size was 4684 surveyed, this calculated based on a difference of 2% (for a social/altruistic motivation that differed by 40% those who had a good achievement and 38% who did not have a good achievement), with a 95% confidence intervals (95%CI), a power of 80% and for a single sample. This was calculated with the formula in Stata: sampsi 0.40 0.38, p (0.8) onesample. The sampling that was used was for convenience, this due to the fundamental objective of finding an association between the main variables of our study. Moreover, an analysis was made of the excluded group according to their general characteristics (sex, age and year of studies), finding no differences.

### Questionnaire and variables

We used Stata version 11.1 (StataCorp LP, College Station, TX, USA) to calculate the sample size through a pilot study. During the sampling stage, we used a 95% confidence level and a statistical power of 80%.

The “General Achievement Motivation Attribution Scale” test [15] was used to evaluate students’ academic performance. This scale is conformed by 18 questions distributed in the following dimensions: attribution to the assignment’s characteristics, attribution to the effort, attribution to the capability and, attribution to the professor’s evaluation. For this test, the results are placed on a Likert scale from 1 to 6 points. We added the total answers—taking into consideration the negative score of questions number 4, 7, 9, 11, 14, 16, and 18 from the General Achievement Scale—and then, the results were divided into tertiles. The superior tertile was categorized as good achievement motivation, in comparison with the ones who did not get a good motivation (the two inferior tertiles). In summary, we considered the students with a good perception of the general academic performance to those who were in the top tertile of all the answers (being contrasted by the students who were in the other two tertiles).

For the variables of the social/altruist and economical/prestige motivations, the “Medical motivation Scale” (MEM-12) was used [16]. This test measures both motivational aspects for choosing the medical career. Each aspect consisted of six questions with five possible answers on a Likert scale. Then, the scores of the answers of each question were added and the totals were divided in terciles, choosing in every case the superior tercile as the ones who had a higher motivation in comparison to the ones who did not (the two inferior tertiles).

The sociodemographic variables were: sex, age, country of origin, years studying, type of university (public or private), family members who are physicians (yes or no). Additionally, the following variables were included in the model: influence on the social media, family pressure, friendship’s influence at school, school grades, economic sources, and vocational tests positive for medical career.

### Ethical considerations

The project was approved by the ethics committee of the Hospital Nacional Docente Madre Niño “San Bartolomé” (ISI CODE: 16020). The participants were informed about the study. Participants were assured about the confidentiality of their data. The questionnaires were anonymous and self-administered. The principles of the Declaration of Helsinki for research in humans were respected.

### Statistical analysis

The statistical analysis was elaborated in Stata version 11.1 (StataCorp LP, College Station, TX, USA). For descriptive analysis, frequencies and percentages of the qualitative variables were obtained, as well as the median and interquartile range of the quantitative variables. For the analytical statistics, the prevalence ratio (PR) with 95%CI and p-values were reported. Generalised linear models, with the use of the Poisson family, log linkage function, thick models, were constructed and adjusted by setting study sites as a cluster group.

For multivariate analysis, we enter all the variables evaluated in the bivariate model, this to serve as an adjustment for the main variable (considering that they are very important in the relationship between the variables of motivation to study the career of medicine and the academic performance).

The outcome variable is the perception of good academic performance in general (category of interest: higher tertile of the total scores of the "General Achievement Motivation Attribution Scale”. These were named as the best perception of academic performance in general. This was adjusted by economical/prestige motivation (category of interest: higher tertile of the scores only of the questions that evaluated the economical/prestige motivation), by the social / altruistic motivation (category of interest: superior tertile of the scores only of the questions that evaluated the social/altruistic motivation) and for the other variables.

## Results

From the 4615 students surveyed, 325 were excluded due to incomplete/blank answers, which left 4290 questionnaires. A total of 2145 (50%) of the students surveyed were females, the median age was 21 years old (interquartile range: 19–22 years old). The 80% of the surveyed belonged to a national/public university. **See** Table 1.

**Table 1:** Social-educative features of medical students in ten Latin American countries.

Variables	N	%
Gender		
**Females**	2145	50.0
**Males**	2145	50.0
Age (years)*	21	19–22
Country		
**Venezuela**	460	9.9
**Honduras**	461	10.1
**Panama**	408	8.9
**Peru**	460	9.9
**Colombia**	460	9.9
**Argentina**	460	9.9
**Ecuador**	463	10.1
**Paraguay**	461	9.9
**Bolivia**	469	10.2
**Mexico**	513	11.2
Type of university		
**Particular/private**	921	20.0
**National/public**	3694	80.0

* Interquartile range.

**Fig 1:**
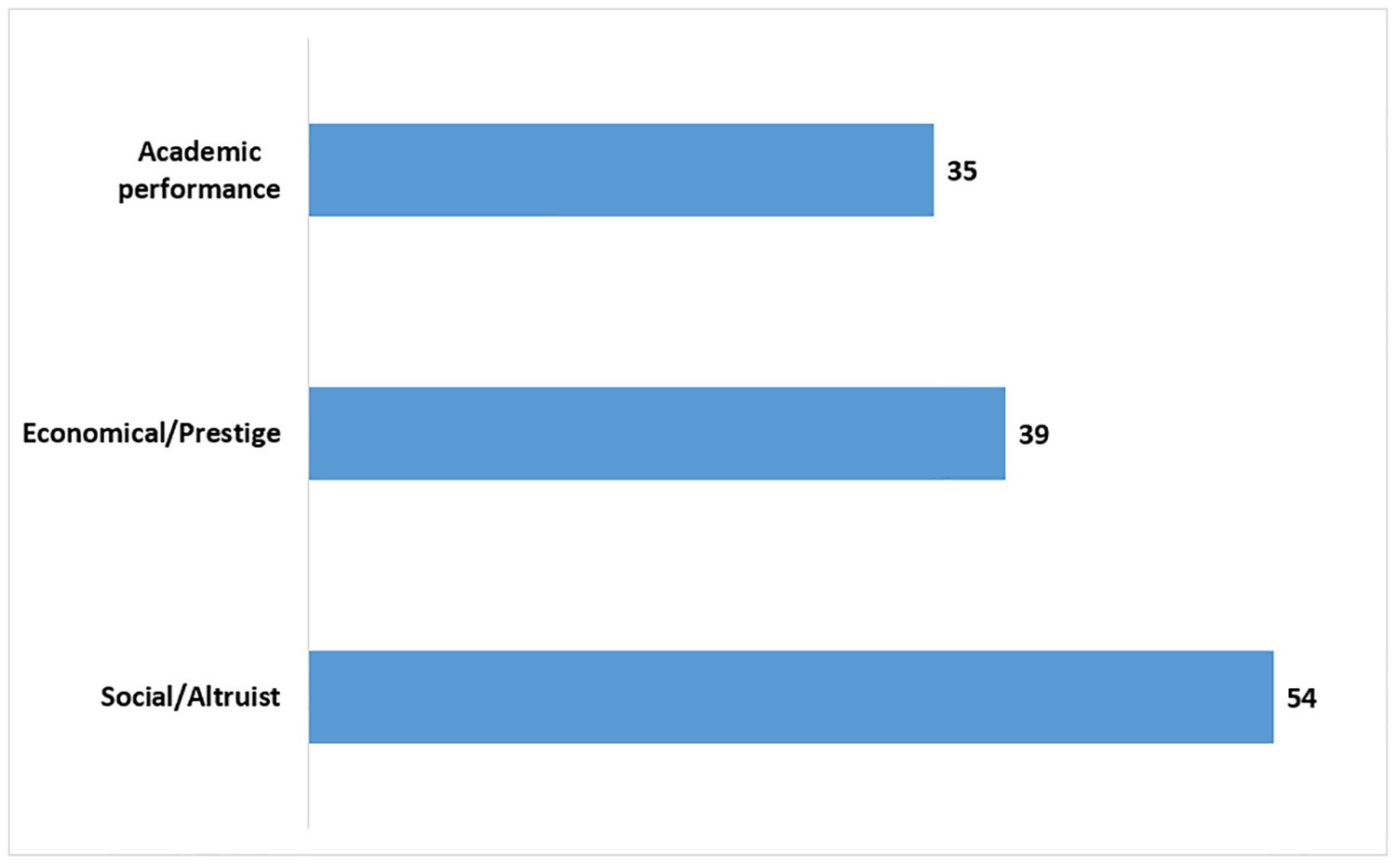
Percentage of motivations types of medical students from ten Latin American countries.

Fig 1 shows that within the studied motivations, the social/altruist motivation represented the greater percentage (54%), followed by the economical/prestige motivation (39%) and the academic performance motivation (35%).

In the bivariate analysis, social/altruist motivation was associated with sex, age, familial pressure, positive vocational test, and good grades at school. The economical/prestige motivation was associated with the age, familial pressure, positive vocational test for a medical career, and good grades at school. A good academic performance motivation was associated with familial pressure, positive vocational test for a medical career, and good grades obtained at school. **See** Table 2.

**Table 2:** Bi-varied analysis of factors associated with the motivation for studying medicine and by general success in medical students of ten Latin American countries.

Variables	Prevalence ratios (95%CI)		
	Social/altruist	Economical/prestige	Good performance
Males	1.17(1.06–1.30)€	0.95(0.83–1.09)	0.87(0.74–1.01)
Age (years)*	0.92(0.89–0.97)£	1.08(1.01–1.17)$	1.05(0.98–1.14)
Private Univ.	1.13(0.37–3.45)	0.75(0.18–3.02)	0.85(0.57–1.28)
Year of study*	1.05(0.92–1.20)	0.98(0.86–1.12)	1.16(0.98–1.39)
Familial pressure	0.13(0.07–0.25)£	2.04(1.24–3.34)€	1.85(1.46–2.33)£
Positive vocational test	3.14(1.58–6.21)€	0.46(0.25–0.84)$	0.69(0.53–0.91)€
Good grades at school	0.56(0.4–0.73)£	2.33(1.17–4.63)$	0.61(0.53–0.69)£

PR (Prevalence ratio), 95%CI (Confidence Intervals at 95%) and *p*-values obtained with generalized lineal models, with *Poisson* family, log linkage function, thick models and taking in count each university as a cluster group.

*Took as a quantitative variable for the analysis.

^$^: p<0.05.

^€^: p<0.01.

^£^: p<0.001.

In the multivariate analysis, male students had a higher social/altruist motivation (PR:1.11,95%CI: 1.03–1.18; p<0,01) and a low motivation for a good academic performance (PR:0.82,95%CI: 0.69–0.96; p<0,05). Those who had familial pressure had a lower social/altruist motivation (PR:0.17,95%CI:0.08–0.36; p<0,001), but higher motivation in academic performance (PR:1.69,95%CI:1.39–2.09; p<0,001) and economical/prestige (PR:1.66,95%CI:1.29–2.13; p<0,001). The positive vocational test was associated with a higher social/altruist motivation (PR:1.85,95%CI:1.03–3.30; p<0,05), but with a low economical/prestige motivation (PR:0.68,95%CI:0.50–0.91; p<0,05) and academic performance (PR:0.73,95%CI:0.62–0.86; p<0,001). Moreover, the good grades at school are related with a higher economical/prestige motivation (PR:1.39,95%CI:1.05–1.83; p<0,05), but lower social/altruist motivation (PR:0.85,95%CI:0.74–0.98; p<0,05) and academic performance (PR:0.63,95%CI:0.50–0.79; p<0,001). **See** Table 3.

**Table 3:** Multivariate analysis of factors associated with the motivation to study medicine and by the academic performance of medical students in ten Latin American countries.

Variables	Prevalence ratio (95%CI)		
	Social/altruist	Economical/prestige	Academic performance
Males	1.11(1.03–1.18)€	Not significant	0.82(0.69–0.96)$
Age (years)*	Not significant	Not significant	Not significant
Private Univ.	Not significant	Not significant	Not significant
Years of study*	Not significant	Not significant	Not significant
Familial Pressure	0.17(0.08–0.36)£	1.66(1.29–2.13)£	1.69(1.39–2.09)£
Positive vocational test	1.85(1.03–3.30)$	0.68(0.50–0.91)$	0.73(0.62–0.86)£
Good grades at school	0.85(0.74–0.98)$	1.39(1.05–1.83)$	0.63(0.50–0.79)£

PR (Prevalence ratio), 95%CI (Confidence intervals at 95%) y *p*-value obtained with generalized lineal models, with *Poisson* family, log linkage function, thick models and taking in count each university as a cluster group.

*Took as a quantitative variable for the analysis.

^$^: p<0.05.

^€^: p<0.01.

^£^: p<0.001.

**Fig 2:**
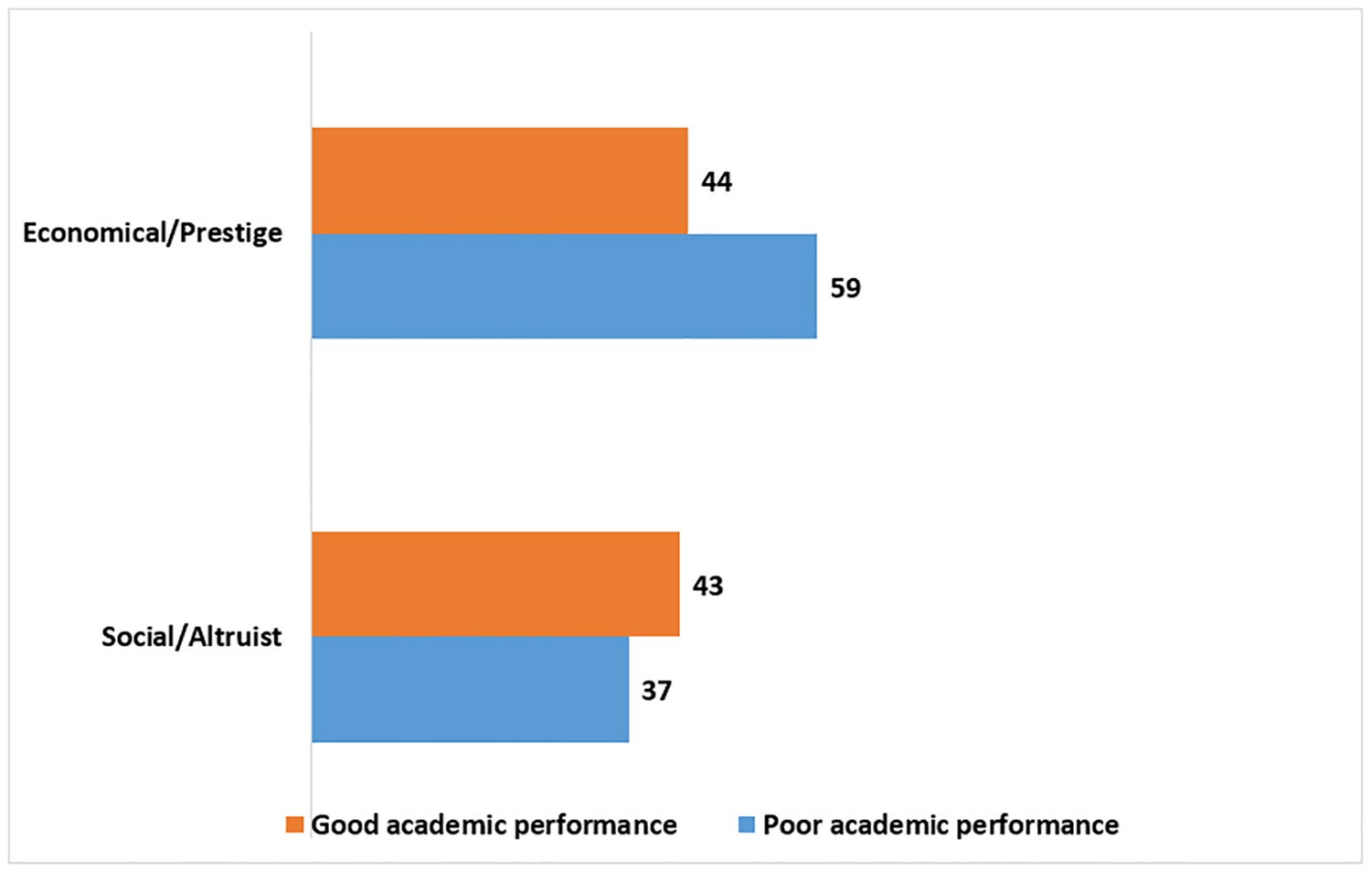
Academic performance (percentage) accorded to the type of motivation of medical students from ten Latin American countries.

Fig 2 shows the statistically significant differences when crossing the academic performance with the social/altruist motivation (p<0.001) and the economical/prestige (p<0.001). Noticing that the ones who had a social/altruist motivation also had a better percentage in academic performance, in comparison to the ones with an economical/prestige motivation.

In the adjusted multivariate model to all the measured variables, we found that a larger frequency of general motivation was related to a less social motivation (PR: 0.57; CI95%: 0.46–0.70; p<0.001), and that it was lower between the male students (PR: 0.83; CI95%: 0.71–0.98; p:0.030), it increased according to the year of study (PR: 1.15; CI95%: 1.03–1.28; p:0.013), was greater when pressure by the family was present (PR: 1.36; CI95%: 1.17–1.59; p<0.001). Furthermore, it was found in a smaller amount in the ones with good grades at school (PR: 0.58; CI95%: 0.45–0.74; p<0.001) and was incremented in the ones that are self-maintained (PR: 1.54; CI95%: 1.26–1.87; p<0.001). Table 4.

**Table 4:** Multivariate analysis of factors associated with the motivation to study medicine and by the academic performance of medical students in ten Latin American countries.

More Motivated	Statistics of association		
	PR	CI95%	p-value
Economical/prestige motivation	1.01	0.90–1.14	0.822
Social/altruist motivation	0.57	0.46–0.70	<0.001
Males	0.83	0.71–0.98	0.030
Age (years)*	1.01	0.97–1.04	0.746
Particular Univ.	0.95	0.82–1.10	0.486
Study years*	1.15	1.03–1.28	0.013
Familial pressure	1.36	1.17–1.59	<0.001
By vocational test	0.84	0.70–1.01	0.062
Good grades at school	0.58	0.45–0.74	<0.001
Self-maintained	1.54	1.26–1.87	<0.001

PR (Prevalence ratio), CI95% (Confidence intervals at 95%) y *p*-value obtained with generalized lineal models, with *Poisson* family, log linkage function, thick models and taking in count each university as a cluster group.

*Took as a quantitative variable for the analysis.

## Discussion

This study showed a higher social/altruist motivation in the medical students Latin America. Our results are similar to some other studies reported [2,7,10,17,18]. In Brazil, freshmen medical students [10] revealed a strong valuation of the humanistic aspects of medicine, a deep personal identification with the choice of profession, and conscious and unconscious desires to help people[10]. In Polish medical students was reported that altruistic and scientific reasons were the main motives for choosing a medical career[2]. Croatian first-year medical students reported lower interest in science and less interest in altruistic aspects of medicine[19]. Yet, Croatian final-year medical students applicants reported even lower interest in science and altruistic aspects of medicine [19]. Moreover, these students would reconsider choosing medicine again because of the corruption in medicine, fear of mistakes and uncertainty of employment [19]. In Chile [16], the main reason for choosing medicine in students from first and seventh year was the social/altruist interest (72.3% and 62.4%, respectively). For medical students in Hungary, the most significant career choice factor also was mainly the altruistic motivation, followed by extrinsic motivations: obtaining a degree, finding a job, accessing career opportunities [7]. Also in Ireland, the social/altruist motivation was an influent factor in students for their decision for choosing medicine [18]. A possible explanation to the decisions taken by students is that the social/altruist motivation appears early in young people with the purpose of helping others, save lives, and contribute to the society [10,19,20].

Male medical students and positive vocational test are related with a higher social/altruist motivation. Conversely, in Polish medical students was reported that mainly the females had an altruistic motivation for choosing a medical career [2]. While the students that indicate familial pressure and good grades at school had a highest economical/prestige motivation, in Malaysia, 77% of students indicated not being distressed by their families [21], moreover another study indicated that coming from a medical family had no influence upon his motivations [6], which is quite different compared with the results of our study. In India, 59% of the surveyed students were forced by their parents to study medicine, while a small percentage of students of 36.8% made the decision by their own [22]. In Cuba, a having a family member working in healthcare is related with the choice of following a medical career, what makes us suppose that influence and pressure increases by having family members who are physicians, specially the parents [23]. On the other hand, in Spain [24], 70% of those surveyed admited the existence of an influential factor in their decision of studying medicine: family pressure (1%), contact with a disease (15%), having family members who are physicians (12%), television (11%), tutors (10%), and friends (5%). Moreover, other explanations were that medical students revealed the the desire to have power, control, knowledge (6) or social status had influenced their decisions [12].

We found strong associations between each form of motivation and academic performance; of note, 44% of students with an economical/prestige motivation had a good academic performance. However, a study in Hungary reported that the lack of altruism was found to be a major risk factor for reduced academic efficacy [7]. In our study, the motivation was the social/altruist type in which a 43% of the students got a good academic performance against a 37% without a good performance. A study reported that selected aspirants for the medical career have greater motivation and better academic performance, in comparison to the students not selected [25]. Our study indicated that medical students’ motivation had a close relation to obtaining a good academic performance. However, is important to note that in many cases motivation is not static and may change throughout a students’ career [13,14,19]. This could be by the results of the socioeconomic influence of their location in which the individual develops, caused by the money the career requires or the wish of getting back the investment made on it.

This study is important because some students Latin America who eventually enter medical school eventually have successful careers. However, some show problems both academic and motivational [19]. On the other hand, a particularly interesting question is whether the sorts of factors we have identified here can also be used either in selecting students, or in counselling them once they are at medical school.

## Conclusion

This study indicated that male medical students and a positive vocational test had the highest social/altruist motivation. Conversely, those who had familial pressure and good grades at school had a lower social/altruist motivation. Moreover, we conclude that medical students’ motivation has a high relation to obtaining a good academic performance, which is vital for the development of this profession.

## Recommendations

Medical schools should fortify the altruist/social motivations through suitable planning, achieving a better organization of the programs, and develop the professional identity training and, therefore, providing adequate opportunities for a better performance of the students.
